# Design and Implementation of a Cloud-IoT-Based Home Energy Management System

**DOI:** 10.3390/s23010176

**Published:** 2022-12-24

**Authors:** Felipe Condon, José M. Martínez, Ali M. Eltamaly, Young-Chon Kim, Mohamed A. Ahmed

**Affiliations:** 1 Department of Electronic Engineering, Universidad Técnica Federico Santa María, Valparaíso 2390123, Chile; 2 Electrical Engineering Department, Faculty of Engineering, Mansoura University, Mansoura 35516, Egypt; 3 Sustainable Energy Technologies Center, King Saud University, Riyadh 11421, Saudi Arabia; 4 Department of Computer Engineering, Jeonbuk National University, Jeonju 561-756, Republic of Korea

**Keywords:** home energy management system, smart home, Internet of Things, cloud infrastructure

## Abstract

The advances in the Internet of Things (IoT) and cloud computing opened new opportunities for developing various smart grid applications and services. The rapidly increasing adoption of IoT devices has enabled the development of applications and solutions to manage energy consumption efficiently. This work presents the design and implementation of a home energy management system (HEMS), which allows collecting and storing energy consumption data from appliances and the main load of the home. Two scenarios are designed and implemented: a local HEMS isolated from the Internet and relies on its processing and storage duties using an edge device and a Cloud HEMS using AWS IoT Core to manage incoming data messages and provide data-driven services and applications. A testbed was carried out in a real house in the city of Valparaiso, Chile, over a one-year period, where four appliances were used to collect energy consumption using smart plugs, as well as collecting the main energy load of the house through a data logger acting as a smart meter. To the best of our knowledge, this is the first electrical energy dataset with a 10-second sampling rate from a real household in Valparaiso, Chile. Results show that both implementations perform the baseline tasks (collecting, storing, and controlling) for a HEMS. This work contributes by providing a detailed technical implementation of HEMS that enables researchers and engineers to develop and implement HEMS solutions to support different smart home applications.

## 1. Introduction

Nowadays, the applications of the Internet of Things (IoT) are appearing in different domains, such as transportation [1], healthcare [2], agriculture [3], and power systems [4]. These IoT solutions aim to monitor and control various elements and devices in different scenarios that will ease tasks and provide useful applications for daily living [5]. In the electric power system, energy plays a central role in powering our homes and appliances. However, the metering process for estimating the consumption of a house is widely dependent on an electromechanical energy meter, which implies that utility companies have to employ personnel to perform the metering tasks monthly in order to bill their customers [6]. As for the appliance consumption within a house, residents may not be aware of individual power consumption for each appliance, therefore facing inefficient energy usage without even knowing. In this regard, Chile has set a goal to have around 6.5 million smart meters installed by 2025 [7]. This goal focuses mainly on using smart meters to lower energy demand by providing more detailed energy billing and interfaces of energy consumption through web interfaces and applications, as well as implementing new tariff systems.

IoT will play a key role in enabling several energy efficiency mechanisms, such as the Internet of Energy, smart grids, and smart homes. This is possible by using digital sensors and communication devices that enable a home energy management system (HEMS), which allows continuous consumption monitoring and appliance control, as well as supporting the communication between the utility and the power grid [8]. Data are collected using IoT devices and later transferred to the cloud-based system infrastructure from where it is stored and processed [9]. Data-driven applications, databases, and file storage systems are key features that can be designed and deployed in cloud-based infrastructure to support the IoT cloud-based requirement for several energy Internet applications.

The design and implementation of a proper architecture are the main challenges for enabling applications based on IoT-collected data on a global level. Several works have proposed HEMS-IoT architectures to solve these challenges. The common criterion used to define an architecture is the data processing and storage location. Three layers are found in the literature where data processing and storage can occur: edge, fog, and cloud.

In this paper, an overview of the HEMS-related work is presented, focusing on key elements, such as the design of a HEMS architecture enabled by IoT devices and the usage of local and cloud computing for data storage and processing. We propose a Cloud-IoT based home energy management system, which helps residents, landlords, researchers, and administrators manage the energy consumption within a house. The proposed HEMS implements a four-layer architecture, which is capable of collecting and storing energy consumption data. Consumption data are obtained from two kinds of devices: smart meters and smart plugs. The smart meter units are able to collect the energy consumption of the entire house, while smart plugs are capable of collecting energy consumption and controlling the power supplied to a single appliance. Two implementations were carried out following two different approaches. In one approach, a local HEMS was isolated from the Internet with a central processing unit. In the other approach, a cloud-based implementation used the cloud for data storage and processing. Both systems provide baseline features, such as collecting measurements from the devices, storing them in a database or performing load control actions, such as turning on/off a device manually or by scheduling. Results show the capability of both systems to perform the collection and storing features for energy consumption and to control the appliances by using smart plugs. The main contributions of this work can be summarized as follows:

- Design and implementation of a four-layer HEMS architecture that allows collecting and storing energy consumption data from appliances and the main load of the home.
- Two scenarios are designed and implemented: a local HEMS isolated from the Internet that relies on its processing and storage duties using an edge device and a Cloud HEMS using AWS IoT.
- To the best of the authors knowledge, this work is the first electrical energy dataset with a 10-second sampling rate from a real household in the city of Valparaiso, Chile, over a one-year period.
- Detailed technical implementation of HEMS will enable researchers and engineers to develop and implement HEMS solutions to support different smart home applications.

The rest of the paper is structured as follows: In Section 2, related work for HEMS is explained. Section 3 presents the proposed HEMS architecture. Section 4, presents two case studies: a local HEMS, and a Cloud HEMS. Section 5 elaborates on the technical details for the implementation of both systems. Section 6 discusses the results from the case studies and the future challenges for HEMS implementations. Finally, Section 7 concludes the paper.

## 2. Related Work

Several applications benefit from energy consumption data obtained from HEMS and appliance control. One example is peer-to-peer (P2P) energy trading which flips the traditional scenario, where electricity is transmitted from large-scale generators to consumers over long distances, while the cash flow goes in the opposite way. In contrast, P2P energy trading encourages multi-directional trading within a local geographical area [10]. With the increasing connection of distributed energy resources (DER), traditional energy consumers are becoming prosumers who can consume and generate energy [11].

Authors in [12] analyzed several IoT applications for smart grids, such as smart homes, smart metering, and energy management, among others. Challenges, issues, and future research regarding the use of IoT to enable Energy Internet (EI) applications were also discussed. A smart home incorporates various IoT-based smart technologies with the goal of providing security, convenience, comfort, energy efficiency, and entertainment which results in improving the quality of life within a residence. Ambient assisted living service, smart energy management technology service, and security are the predominant technology services associated with smart homes [13].

In [14], the authors presented an overview of IoT-enabled energy systems. Some of the outlined challenges include mapping every object into a unique virtual object which can be addressed with standard communication protocols. The authors also stated that given the variety of design decisions made by the system designers, there are different architectures to enable an IoT-based energy system, which implies that there is no unified architecture. In [15], the authors presented a smart load node (SLN) for enabling non-smart home appliances to operate efficiently in a smart grid paradigm. SLN is an innovative solution given that it does not require any modifications in the electrical wiring of a house, nor any modification on the appliances. SLN integrates within a HAN with other devices, such as smart meters and a load management unit (LMU), which enable various smart grid applications within a house, such as scheduling loads in a demand–response (DR) scheme. Authors in [16] presented a novel methodology including the concept of green building in order to reduce energy consumption. A key element stated by the authors is not only regarding the energy efficiency for appliances and at home but also to create awareness among residents on power conservation.

Authors in [8] presented a survey on HEMS which provides an aggregated and unified perspective on residential buildings. An overview of the literature on commonly managed household appliances was also presented. Home energy management systems (HEMS) aim to improve efficiency by providing control of smart home appliances, and this is feasible due to the use of the Internet of Things (IoT) [8]. HEMS relies on smart sensors, appliances, and advanced metering infrastructure (AMI) to achieve continuous monitoring. Authors in [16] presented a survey on the concepts, technical background, architecture, and infrastructure, among other challenges and issues regarding HEMS. The use of IoT will allow any smart device, also known as the “things” to interact with one or several sensors and other devices in the network, forming a wireless sensor network (WSN). This WSN can rely on a gateway for Internet connection, allowing the implementation of applications based on the collected data. Authors in [9] presented an overview, architecture, and implementation of IoT in energy systems. The HEMS follows some baseline features, such as monitoring of the main load of the property, individual loads of appliances, and control of appliances. Regarding the components that comprise a HEMS, such a system possesses sensing and measuring devices, smart appliances, a user interface, and a central platform.

Due to the services provided by public clouds, there has been an increasing interest in developing data-driven applications. Some of the data-driven applications that can be implemented in the context of a smart home are alarms on irregular load scenarios and scheduling the use of appliances in case of dynamic tariff systems. In [17], the authors presented a comparison between three cloud platforms: Amazon, Google, and Microsoft. MQTT messaging was used by IoT devices to send information to the cloud platforms, where a performance evaluation was carried out, not to benchmark the maximum message throughput, but rather to measure the service time of the provided message broker. Cost comparison and description of available tiers were also discussed.

Authors in [18] developed a demand response (DR) application on a HEMS in order to reduce utility operational costs and the consumer energy bill price. The proposed infrastructure by the authors is based on an edge–fog–cloud computing architecture, which allows for monitoring and control of residential loads. The testbed was carried out using Raspberry Pi as the HEMS and NodeMCU ESP8266 as smart plugs for energy-related measurements and controlling tasks. Results showed that the proposed system was able to schedule loads and reduce the energy bill when compared to the scenario without the DR algorithm. The proposed testbed scenario considered a dynamic tariff system. Another energy management system (EMS) was proposed by authors in [19], where a system was implemented at the IoT Microgrid Laboratory at Aalborg University. The IoT-based EMS showed the feasibility of using IoT devices to regulate consumption. Features, such as energy management using load priority, were presented in the results.

Authors in [20] presented a cloud-based platform that collects electricity consumption, indoor climate, and occupancy data in real-time using sensors. The energy monitoring platform was implemented in a smart villa. The architecture showed the devices’ interaction over a star topology. The system utilizes ThinkEE, a cloud platform for connecting IoT devices. It also provided a web interface for data display and an energy management system for energy control. Authors in [21] described the building operation data, which includes electricity consumption and environmental measurements. The work provided information regarding the architecture of the system, which utilizes EMU, smart meters, and sensors for collecting the data. The data include one-minute interval measurements from 1 July 2018 to 31 December 2019 which are provided to support a variety of data-driven applications. In [22], the authors released I-BLEND, a 52-month electrical energy dataset at a one-minute sampling rate from commercial and residential buildings of an academic institute campus. The data collection of the system was carried out using a Raspberry Pi to collect measurements from smart meters, while also using the cloud for data storage and processing. The authors in [23], introduced Plug-Mate, an IoT-based occupancy-driven plug load management system. Plug-Mate was able to deliver occupancy information, plug load type, and plug load usage preference. The solution was tested during a 5-month study in a university office with 10 participants. Results showed about 51.7% in the overall energy saving improvement among different plug loads and about 7.5% reduction in the building overall energy consumption.

Although previous research provided information on how to implement IoT-based HEMS and how to apply data-driven algorithms, such as demand response and load schedule, most of the solutions were implemented in a laboratory environment that does not represent the actual condition of a smart home. This work aims to fill the knowledge gap by providing a detailed description of the technical implementation of how to design and implement a HEMS that can be used for different applications. Two architectures are considered for local/cloud implementation using available IoT devices while deploying the system on a public cloud. Table 1 shows the comparison among previous research work.

## 3. Cloud-IoT Home Energy Management System

**Figure 1:**
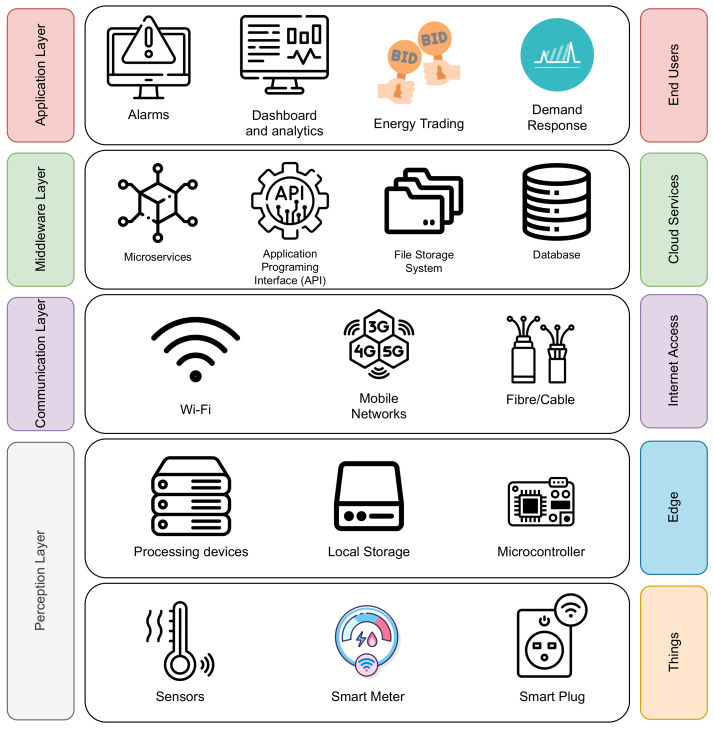
Cloud-IoT HEMS architecture.

Figure 1 shows the proposed Cloud-IoT HEMS architecture. It consists of four layers: perception layer, communication layer, middleware layer, and application layer.

### 3.1. Perception Layer

The perception layer enables data collection through sensors, as well as data storage and data processing tasks through edge devices. This layer considers several physical devices, such as sensors, smart meters, and smart plugs, which are often referred to as “things”. Edge devices enable tasks, such as data storage, data processing, and performing actions, on things that are located in the edge sub-layer. The perception layer is a key element in the proposed architecture, working as the first step in the energy data collecting process and as the last step in the appliance controlling process.

- Things Layer: The things layer is composed of sensors and actuators. Sensors are used to collect information on important measurements of the smart home. Sensors, such as temperature, humidity, and light detection, among others, are used for comfort purposes. Regarding appliances’ power consumption, two devices are introduced: smart meters and smart plugs. A smart meter is able to collect energy consumption information of the main loads of the smart home. It utilizes a current transformer (CT) and a voltage sensor to compute the power consumption. Smart plugs (SP) work as a middleware between the power line of the house and the appliance plug. The purpose of SPs is to collect energy consumption data from a specific appliance while also being able to control the energy delivered to the appliance using a relay.
- Edge Layer: The edge is the closest to the sensors and actuators. It provides the capability to collect data and perform command operations over “things”. The edge layer considers a low latency but limited data storage and processing capabilities as available resources. From a HEMS perspective, the edge layer is found at the house level, where it is able to use this layer as a perception layer to collect the data of interest.

### 3.2. Communication Layer

This layer allows the connection of the perception layer with the middleware layer. Several communications technologies could be used to perform this task, among them WiFi, Zigbee, LoRa, Fiber, and mobile networks such as 4G and 5G. The election of the technology has various attributes, such as effective range (short range and long range), cost, coverage, and availability of a given communication system. An important role of the network layer is providing a home area network (HAN) for perception layer devices to join. The HAN allows communication between devices, also known as machine-to-machine (M2M) communication, which allows routing the data from the things to the edge or from the edge to the middleware layer.

### 3.3. Middleware Layer

The middleware layer is related to cloud-based services that rely on the received data to perform some tasks. The most common functions of a HEMS consider storing measurements in a database, performing data processing tasks through microservices, and providing an application programming interface (API) for managing data requests. These services can be implemented by using a public cloud, such as Amazon Web Services (AWS), Google Cloud Platform (GCP), and Microsoft Azure (Azure). Given the functionality, the middleware layer is similar to the edge layer but provides a scalable infrastructure to serve higher traffic and more demanding processing and storage tasks. Several technology stacks are available for developers to carry out the required implementation to achieve the abovementioned tasks. Some challenges and decisions that need to be addressed in order to provide a robust system consider database election, designing and deploying the required cloud architecture, programming language, and/or framework election. The middleware in this architecture serves as a bridge between the data perspective provided by the “things” and the intended smart home features or applications identified in the application layer.

### 3.4. Application Layer

A HEMS aims to provide data to several domains, such as Energy Internet, smart grids, and smart homes. Some data-driven applications for HEMS are, for example, demand response, P2P energy trading, and monitoring energy consumption for user awareness. These applications serve as the last layer in the proposed architecture which is the closest to the end user.

## 4. Home Energy Management System Design

Two case studies are proposed to compare different approaches for HEMS: a local HEMS and a cloud-based HEMS. On the one hand, a local HEMS system uses a central computing unit to handle data storage and processing tasks. On the other hand, a cloud-based system collects the data through a gateway and is able to enable several solutions and applications.

Both systems are designed to meet the following tasks, which enable HEMS to perform some features of a smart home:

- Monitoring of the energy consumption: HEMS should store data about the power consumption of the appliances monitored by the smart plugs and the main load of the smart home.
- Appliance control: HEMS should allow a resident to interact with the appliances connected to smart plugs to supply or deny energy.

In this work, the smart home includes some features that are present on both systems as a requirement. We recognize two categories that are common ground for the implementation of such systems:

- The things: Both case studies utilize the same end devices: smart plugs and smart meters. The task of the smart meter is to collect the total power consumption reading of the house. On the other hand, a smart plug is able to collect the power consumption and control the supplied power of a single appliance.
- Networking and communications: From a communication perspective, we considered that the smart home has WiFi capabilities, enabling devices to interact within a home area network (HAN). Devices such as smart plugs and smart meters have the capability to join the HAN using WiFi technology. An Internet Protocol (IP) address is assigned to each device that joins the network. The things have the capability to periodically send measurements of the energy consumption telemetry to the destination. Direct energy consumption requests can also be performed, following the messaging protocol that each device supports. The things send the collected data using MQTT, a popular Pub/Sub (publisher/subscriber) messaging protocol, where each device sends data over a unique topic.

**Figure 2:**
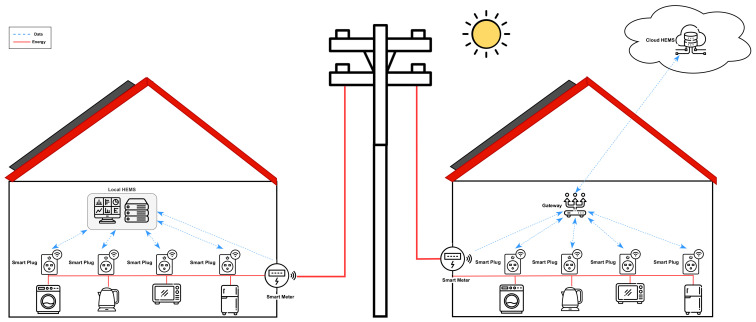
Schematic diagram for HEMS. **Left**: Local HEMS design, **Right**: Cloud HEMS design.

Figure 2 shows a schematic diagram for HEMS design. On the left side, the system is composed of home appliances, a smart meter, smart plugs, and a computing unit serving as the local HEMS. This approach considers a solution that relies solely on monitoring and managing house appliance consumption in a local scenario. Such solution presents benefits from a privacy perspective, given that it is isolated from the Internet. The main drawback of this kind of approach is the limited computational and storage capacity of the HEMS, which is directly related to the provisioned on-premise hardware. From the Edge perspective, the devices such as smart plugs and smart meters are intended to collect energy measurements based on the energy consumption of certain appliances and the total energy consumption of the main home, respectively.

The design of the Cloud-IoT HEMS is presented on the right side of Figure 2, where the energy and data interactions are shown. Smart meters and smart plugs allow the collection of energy-related data. The main objective is to send the energy consumption data to the cloud. For this purpose, the things send information to a gateway, which allows bridging the data into the cloud.

These data are stored and processed by databases and cloud microservices. Several data-driven HEMS applications can be designed and implemented. One benefit of using public cloud services is the infinite scaling opportunity, which allows applications to scale as needed. Cloud providers manage the hardware and configurations required to enable any architecture to function appropriately, easing the development experience. This allows for cheaper costs when comparing cloud-based implementations to on-premise implementations for a scalable system.

## 5. Implementation

The testbed was carried out in a real house located in the city of Valparaiso, Chile. A wireless local area network (WLAN) was set up by a router that provides WiFi connectivity for all devices located in the house. The energy consumption data was acquired starting from July 2021 up to September 2022. Four appliances were used: a kettle, a washing machine, a refrigerator, and a microwave.

**Figure 3:**
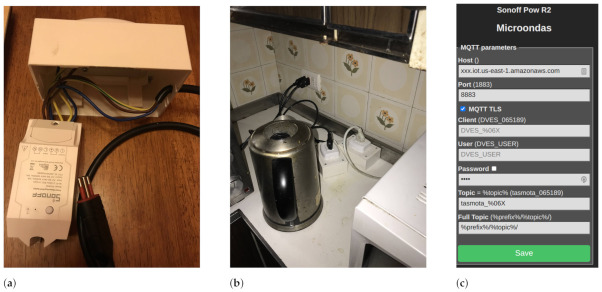
Sonoff POW R2 as smart plug, overview, usage, and configuration. (**a**) Smart plug unit; (**b**) Two smart plug units connected to a kettle (**left**) and a microwave (**right**); (**c**) MQTT configuration on Tasmota web interface.

The selected smart plugs were the Sonoff POW R2, which were modified to fit inside a small electrical box, as seen in Figure 3a. This configuration provides one outlet for connecting the appliance and one plug for connecting the unit to the power outlet of the home. Each appliance was connected to a smart plug, as seen in Figure 3b. These devices were configured using the open-source firmware Tasmota. This firmware allows for an easier development experience enabling easier management of the configurations of smart plugs. The web interface was used to set up the WiFi connection with the HAN.

On the smart plugs, MQTT was set using the web user interface (UI) by specifying the required connection parameters, as seen in Figure 3c. Each smart plug uses a unique MQTT topic to publish and/or subscribe to messages. The Sonoff devices were configured to send one measurement every 10 s using the telemetry feature within Tasmota. Message data were transmitted using JavaScript Object Notation (JSON) structure. The topic structure for smart plugs is “tele/device-ID/sensor”, which represents the telemetry event sent by a specific device informing its sensors data collection.

**Figure 4:**
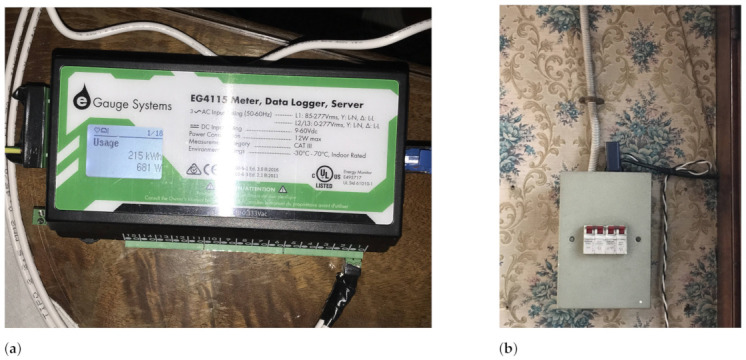
eGauge EG4115 data logger used as smart meter to collect energy consumption data from the main load of the house.(**a**) eGauge data logger unit showing instant power consumption; (**b**) CT Sensor (blue device) used to collect the current of the main load of the house.

For the smart meter, the eGauge EG4115 unit was selected, as seen in Figure 4a. This device uses current transformer (CT) sensors, as seen in Figure 4b, and voltage sensors to compute the energy consumption of the house with frequencies of one measurement per second. This device was connected via Ethernet to the router, joining the local area network (LAN) and obtaining an IP address. This device comes with XML API within its firmware, which enables any device in the network to request measurements of the unit.

Messages sent by smart plugs and the smart meter are composed of several attributes that compose the payload of the message. Table 2 presents the data structured object provided on the event emission by the smart plug and the smart meter. These messages are structured in a data object based on a key-value pair structure. The attribute column in the table references the key of the data object, while the type column provides the data type of the value associated with the attribute. The description and measuring unit are provided for all attributes on the smart plug and the smart meter.

### 5.1. HEMS Local Implementation

**Figure 5:**
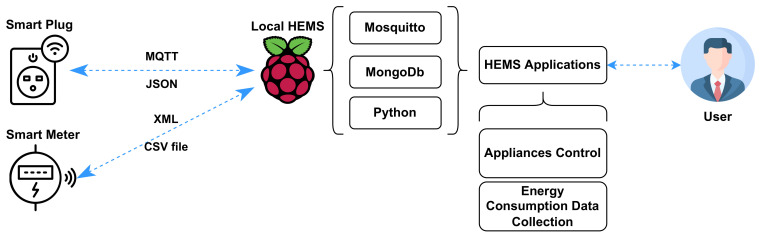
Schematic diagram for local HEMS implementation.

The local implementation was carried out using a Raspberry Pi as the local HEMS, as shown in Figure 5. A Raspberry Pi was configured with Raspberry Pi OS-Lite, which is a Linux distribution developed to serve as the suggested operating system (OS).

Several services were configured in the Raspberry Pi to allow this unit to establish MQTT communication, provide a database to store measurements, and perform lightweight processing tasks. Mosquitto is an open-source MQTT broker, which was used in the Raspberry Pi to participate in a publish–subscribe messaging scheme with the things. The MQTT broker was set up on port 1883.

MongoDB is a non-relational database that allows storing data in a JSON structure. A single MongoDB instance was set up on the Raspberry Pi on port 27017. Python is a high-level scripting language that was used to automatize the process of storing a document in the database for each measurement received. Three scripts were developed: the first one for collecting the measurements of the smart plugs, the second one to collect measurements of the smart meter, and the last one to handle appliance control commands. The first script uses the paho MQTT client, which connects to the MQTT broker and subscribes to each smart plug MQTT topic. PyMongo, a MongoDB client, is used to perform operations in the database. For each message received by the broker, the content of the message is saved into the database.

For collecting the smart meter measurements, the second Python script uses requests, an HTTP client for Python, to request the last four hours of energy consumption recorded by the eGauge unit. This request is fulfilled by a CSV file that contains the requested data. The file is saved in local storage within the Raspberry Pi. This script is scheduled to be performed every four hours using a cron job. Please note that the time of “4 h” could be adjusted based on application requirements. The last script provides the capability to send commands to a specific appliance topic for turning the relay of the smart plugs ON or OFF.

### 5.2. HEMS Cloud-Based Implementation

**Figure 6:**
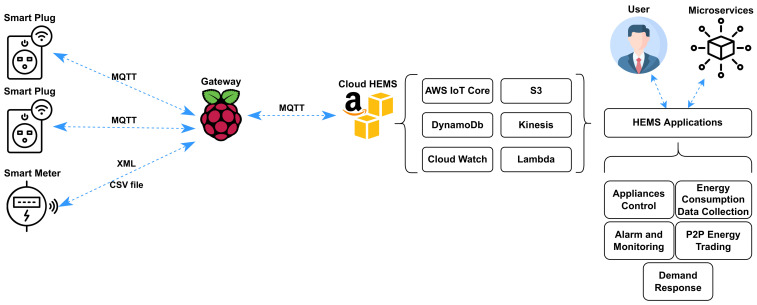
Schematic diagram for Cloud HEMS implementation.

The Cloud HEMS system was implemented following the structure presented in Figure 6. A Raspberry Pi was configured as an MQTT broker using mosquitto. This broker was used as a host by the things to send the telemetry events using MQTT on individual topics. The MQTT broker was set up in a bridge mode, allowing it to route the inbound data to a new destination provided by the cloud provider.

**Figure 7:**
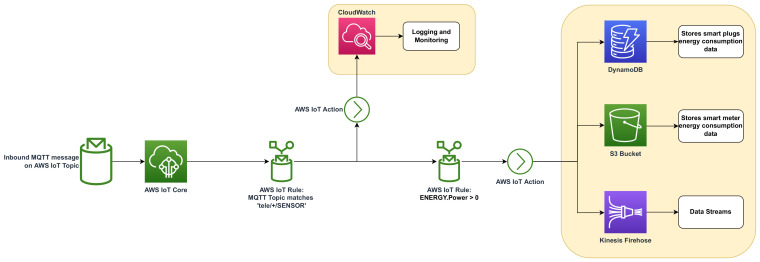
AWS IoT Core implemented rules and actions.

AWS IoT Core was set up using the AWS console. The IoT Core provides an MQTT broker, which is able to manage MQTT connections from smart homes. IoT Core also allows setting up triggers for executing events when data is received. The implemented triggers and actions are presented in Figure 7.

Two rules are set in order to perform some actions. The first one considers that all the incoming messages that match the topic structure “tele/+/sensor” will be logged into AWS CloudWatch. The plus sign “+” is an MQTT single-level wildcard on AWS IoT Core rules that matches any value in that position; in this case, it represents the device ID from the smart plug unit. The rest of the actions are executed only when the second rule is met, which implies that the power consumption informed by the received message is greater than zero. This rule was set to optimize the storage and costs of the implemented system. When the rules are not matched by the incoming MQTT messages, these are discarded. Regarding the actions, the Cloud HEMS stores the data using DynamoDB, where smart plug data are stored in a JSON object using the schema and attributes presented in the data payload in Table 2. For storing the smart meter data, an S3 bucket is used to handle the CSV files provided by the unit over MQTT. Kinesis Firehouse is used to ingest the received MQTT message into a data stream enabling further processing, data events, and real-time-based applications.

### 5.3. Case Study 1: Local HEMS Results

**Figure 8:**
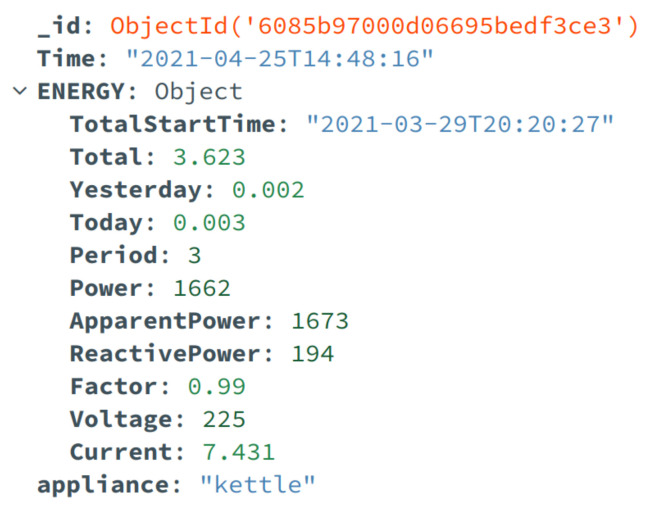
Database record of smart plug measurement of the kettle.

For the local HEMS, results show the capability of the system to store the energy consumption data of the appliances and from the main load of the house. Figure 8 shows a snapshot of the MongoDB collection that contains the measurements from the appliances collected by the smart plugs.

**Figure A1:**
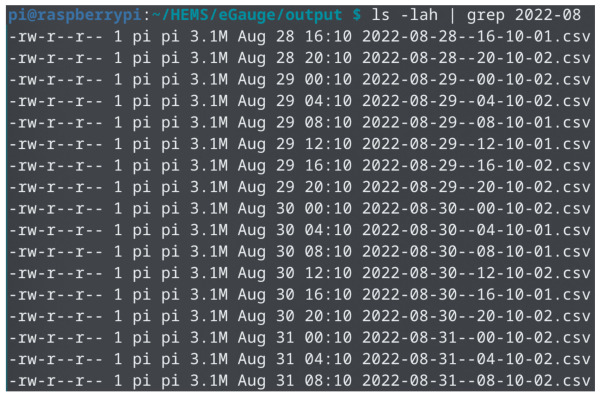
Smart Meter energy records from eGauge unit.

Each document contains the energy-related parameters for each telemetry event. The document contains one parameter that allows for appliance identification. By using the ID of the smart plug, it is labeled as the correct appliance with the same name attribute in the database document. This parameter is configured in the collection as an index, which allows for faster queries when used as a filter parameter. The records collected by the smart meter are shown in Figure A1, where they are stored as CSV files in a directory.

### 5.4. Case Study 2: Cloud HEMS Results

**Figure A2:**
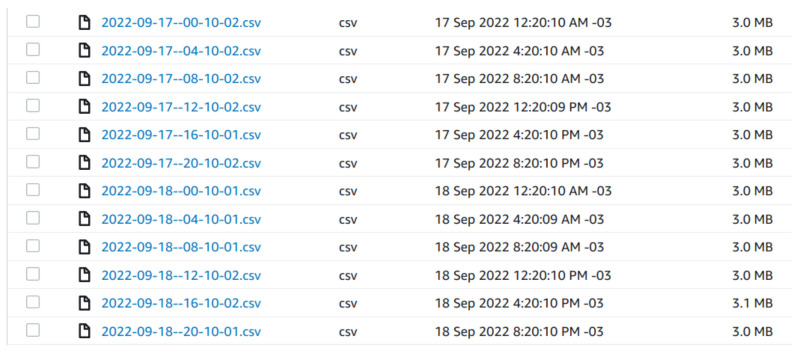
AWS S3 bucket with energy consumption records from smart meter.

Cloud HEMS was deployed starting in July 2021, during which data were collected and stored in the cloud. The AWS S3 web panel allows checking the individual files stored by the system for the smart meter measurements, as seen in Figure A2. Each file is around 3MB, and it contains a four-hour window of measurements taken by the smart meter every second. These files follow the structured data provided by the eGauge unit.

**Figure 9:**
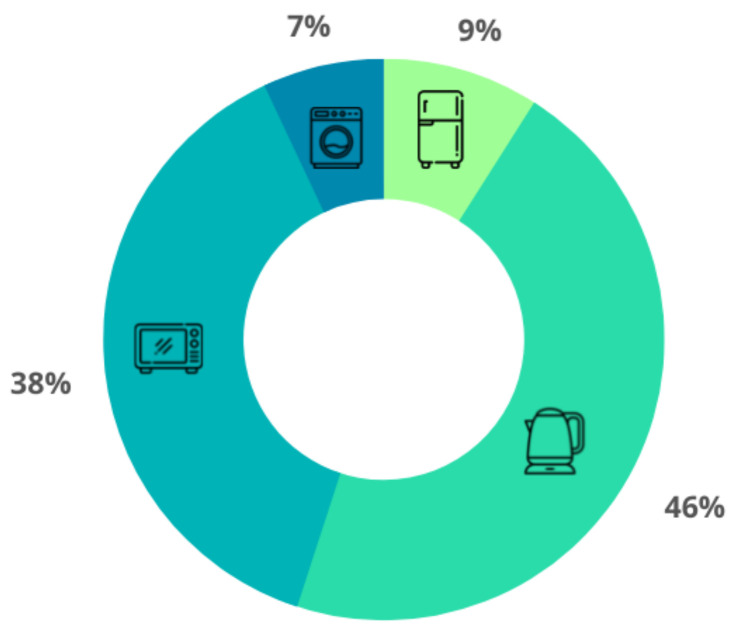
Energy consumption per appliance for resident awareness.

Batch processes can be scheduled to perform analytics operations over the stored data. Figure 9 shows a pie chart for the energy consumption per appliance for the first week of August 2022. This information intends to serve the residents as feedback on their behavior regarding the energy consumption of each appliance.

**Figure A3:**
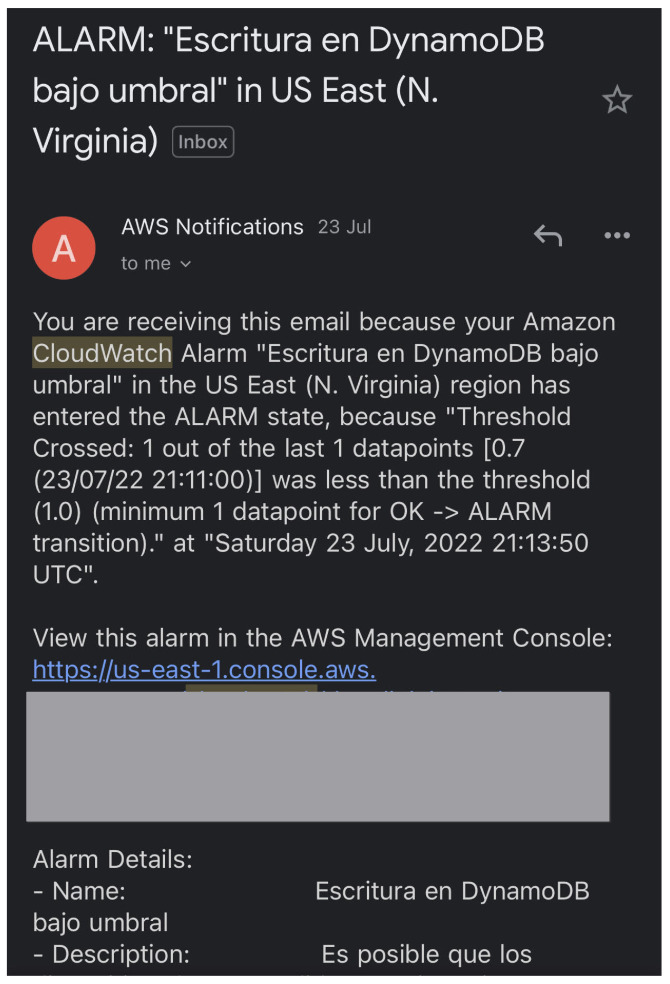
Email notification when a smart plug has not write into the database in a fixed period of time.

One feature that benefits from data analysis services provided by AWS in the cloud is shown in Figure A3, where an email notification alerts when the system has not detected any writing activity in the database. This enables the residents to be aware of appliance malfunction, energy blackouts, connectivity issues, or any other problem regarding the energy consumption in the home.

**Figure 10:**
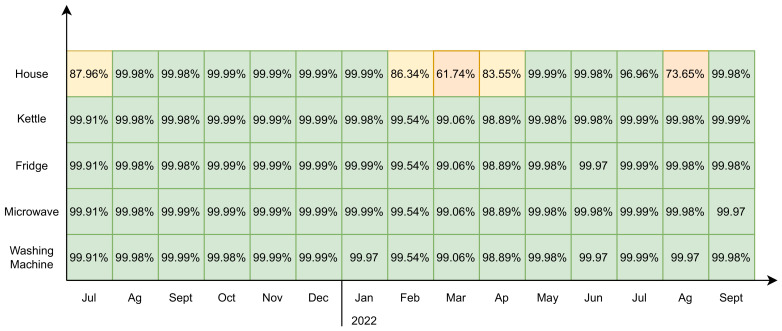
Analysis of system availability for the collected data.

Figure 10 shows the data availability of the system regarding the collected data from the devices. The x-axis presents the available time period since the system started recording data from July 2021 up to the end of the study (grouped by months). The four appliances and the main load of the home are shown on the Y-axis. The values shown for each device and the month represent the percentage number of messages received from each device during a certain month over the total amount of messages that could be received considering the sample rate for each device. This graph shows that in some periods, such as August 2022, there were some constraints on receiving data from the house. This happened due to a system malfunction, blackouts, or scheduled maintenance from the power utility provider.

**Figure 11:**
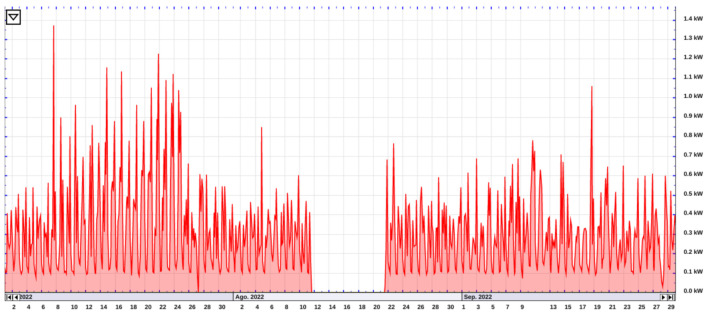
Total power consumption of the main load of the house using the eGauge unit during the period of July, August, and September 2022.

**Figure 12:**
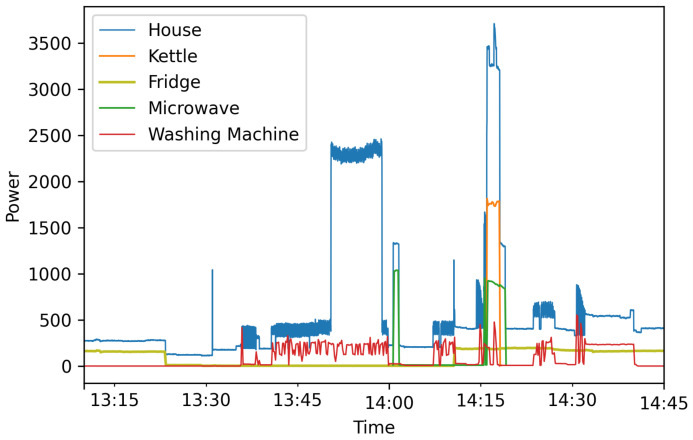
Power consumption data from the four appliances using smart plugs and the main load of the house.

Regarding data visualization, Figure 11 shows the main load of the house collected by the eGauge unit. The period shows the regular power consumption over the month of August with a gap of data. In Figure 12, a window of two hours shows the specific energy consumption for the kettle, refrigerator, microwave, washing machine, and the main load of the house on 2 August 2022. A slice of the data collected from our real house implementation with some instructions regarding the implementation of the HEMS can be found at our repository of the project https://github.com/pipegreyback/EViG-Server, (accessed on 1 October 2022).

## 6. Discussion

Data-driven applications provide an enormous benefit in various fields. In distribution power systems, the new applications of the Energy Internet, smart grids, and smart homes will allow, for example, disaggregated consumption per appliance using the total power consumption of the home. Some other applications allow load scheduling for appliances. These applications require reliable architecture and implementation that allow the collection, storage, and processing of such data. Such a challenge has been discussed widely, but there is no one standardized architecture that fits all solutions, thus the architects and developers need to identify the requirements of the system in order to design a reliable architecture that allows the system to function properly. Some of the considerations will enforce the data storage and processing to be deployed locally at the edge or remotely in the cloud. Nevertheless, a system such as the proposed HEMS could present different requirements when multiple houses implement the system. There are other constraints, such as a massive amount of data sent to the cloud from individual houses, which could result in high latency and degradation of the service. Thus, solutions that use a fog layer as data concentrators within a neighborhood, such as Neighborhood Area Network (NAN) should be considered when looking to deploy a HEMS in various houses within a neighborhood.

New tools such as AWS Cloud Development Kit (CDK) for speeding the provisioning process of services appear as an interesting alternative to provisioning infrastructure through web interfaces, such as the AWS console. AWS CDK follows the paradigm of Infrastructure as Code (IaaC) which enables the developers to write code in several programming languages such as Typescript, Python, and Go. The code represents the required services to be provisioned, with the respective configurations which are then synthesized and deployed into AWS through cloud formation.

### 6.1. Technology Adoption

The acquisition of dedicated sensors and actuators with Internet capabilities and IoT devices provide multiple benefits for different data-driven applications, but it comes with the challenge of technology adoption. In [24,25], the authors investigated the user perception on acceptance of monitoring energy consumption devices such as smart meters. In [24], the authors reviewed consumer beliefs regarding smart meters using behavioral decision research. Results showed that consumers are positively predisposed toward smart meters; however, the authors proposed recommendations for electric utilities in order to address misconceptions about smart meters’ benefits and concerns over risks. Even though the use of smart energy management systems in the residential context provided energy savings, increasing user acceptance has been a challenge over field implementations [25]. The authors identified seven high-level categories based on a mixed-method approach providing a more holistic understanding of users’ perception of smart energy management system adoption.

### 6.2. Implications of the Proposed Solution and Future Directions

The design of HEMS still entails a degree of uncertainty, given that developers and architects might propose different approaches on how to carry out such a system. This is due to multiple factors, such as the variety of IoT-enabled devices, networking and communication protocols, and data resolution requirements. The usage of IoT devices such as smart plugs, a common component of both implementations, provides the benefit of enabling existing households to benefit from smart grid applications without interventions of the property. Our implementation was able to perform a set of features available in HEMS for a real house in Valparaiso, Chile. However, another direction is the integration of other communication protocols such as Zigbee or LoRa. Such solutions will enable our system to support heterogeneous IoT systems, which provide flexibility while choosing IoT end devices such as smart meters and smart plugs.

## 7. Conclusions

In this paper, we designed and implemented two HEMS solutions that are able to store power consumption and control appliances by using edge (local) devices or the cloud. The proposed architecture consists of four layers: perception layer, communication network layer, middleware layer, and application layer. The local HEMS is isolated from the Internet and utilizes an edge device to serve as the main processing unit. The cloud-based HEMS utilizes a gateway to send the data to the cloud. Both implementations are driven by IoT devices to send data measurements or receive control signals. We reviewed, designed, and implemented the most common approaches on state-of-the art edge (local) HEMS and cloud-based HEMS. Both systems have some common features of HEMS; however, they differ in terms of privacy and scalability. In this regard, new challenges appear when multiple HEMSs need to be deployed in a community or a neighborhood area network. A hybrid approach could enable a more reliable and integral solution than using edge devices or the cloud as individual systems. Our ongoing work considers extending the developed HEMS to support different applications, such as energy disaggregation, anomaly detection, demand response, and peer-to-peer energy trading, further extending the system capabilities to enable real-time data processing applications through data streams.

## Figures and Tables

**Table 1:** Comparison among previous research work.

Ref.	Year	Type	Description
[8]	2020	Survey	A survey on home energy management including main goals for operation and target strategies
[9]	2021	Survey	Comprehensive study of IoT business applications and smart energy systems
[10]	2018	Technical/Simulation	P2P energy trading was designed and simulated for energy trading among prosumers and consumers in a microgrid
[11]	2014	Technical/Simulation	Energy trading among prosumers in a microgrid to increase the utilization of renewable energy
[12]	2019	Survey	Comprehensive survey on IoT applications for smart grid and smart environments
[13]	2021	Review	Literature review on smart home adoption including motivations, barriers, and risks
[14]	2018	Review	Review on IoT-based energy system with respect to features, specifications, communication infrastructures, and privacy
[15]	2019	Technical/Implementation	Design and implementation of a low-cost smart load node for monitoring and control non-smart residential load
[16]	2022	Review	Comprehensive review for home energy management system with respect to concepts, architecture infrastructure, and challenges
[17]	2020	Technical/Simulation	Performance analysis among three different Cloud-IoT platforms services for Amazon web service, Microsoft Azure, and Google Cloud
[18]	2021	Technical/Implementation	IoT-based infrastructure on edge–fog–cloud architecture to monitor and control residential loads to support demand response
[19]	2019	Technical/Implementation	IoT-based infrastructure for EMS. The system has been tested in a pilothouse named IoT Microgrid Living Lab, Denmark
[20]	2019	Technical/Implementation	Energy monitoring platform to collect real-time electricity consumption data in a smart villa, Doha Qatar
[21]	2020	Technical/Implementation	Detailed building operation data (electricity consumption and indoor environment) of seven-story building in Bangkok, Thailand
[22]	2019	Technical/Implementation	Electrical energy dataset (52 months) from commercial and residential building at one minute sampling rate, India
[23]	2022	Technical/Implementation	IoT-based plug load management system capable of providing occupancy and energy consumption information for smart building, Singapore
This work	2022	Technical/Implementation	Design and implementation of two HEMS architectures (local vs. cloud) in a real household environment located in Valparaiso, Chile

**Table 2:** The things, smart plug, and smart meter energy data collection attributes on messages.

Device	Attribute	Type	Description	Unit
Sonoff POW R2 Smart Plug	TotalStartTime	S	Starting timestamp for period computations	Date
	Total	N	Total Energy usage including Today	kWh
	Yesterday	N	Total Energy usage between 00:00 and 24:00 yesterday	kWh
	Today	N	Total Energy usage today from 00:00 until now	kWh
	Period	N	Energy usage between previous message and now	Wh
	Power	N	Current effective power load	W
	Apparent Power	N	Power load on the cable	W
	Reactive Power	N	Reactive load	W
	Factor	N	Power factor of the load	-
	Voltage	N	Current line voltage	V
	Current	N	Current line current	A
eGauge EG4115 Smart Meter	Date & Time	N	Timestamp of the current measurement	Unix Timestamp
	Usage	N	Current effective power load	kW
	Current	N	Current line current	A
	Voltage	N	Current Line Voltage	V
	Factor	N	Current Power factor	-

## Data Availability

Not applicable.
